# Neutrophils have altered response to acute respiratory viral infection in sputum of patients with rheumatoid arthritis

**DOI:** 10.1016/j.jacig.2026.100665

**Published:** 2026-02-26

**Authors:** Matthew Lawrance, Naresh Doni Jayavelu, Gabrielle Z. Yize Smith-Rockne, Holly Akilesh, Anne Chaize, Vivian Gersuk, Hannah DeBerg, Stephan Pribitzer, Adarsh Manjunath, Matthew C. Altman, Cate Speake, Jane H. Buckner, Carmen Mikacenic

**Affiliations:** a Benaroya Research Institute, Seattle, Wash; b University of Washington, Division of Allergy and Infectious Diseases, Seattle, Wash

**Keywords:** Neutrophils, sputum, rheumatoid arthritis, airway, transcriptomics

## Abstract

**Background:**

Rheumatoid arthritis (RA) has been shown to pathologically modify the human lung environment. Individuals with RA have been shown to have higher incidence of respiratory infections and worse resultant patient outcomes.

**Objective:**

We investigated whether single-cell transcriptional signals within sputum distinguished healthy lungs from those of patients with RA before and after infection.

**Methods:**

Sputum samples were collected at both baseline (study enrollment) and 1 month following respiratory infection. Expectorated sputum was fixed at time of sampling and sequenced via a 10*x* Flex single-cell RNA sequencing protocol. Cells were clustered via transcriptomic signal, and cell types were identified via canonic markers. Differentially expressed genes within cell types between disease state and timepoints were grouped into coexpressed gene modules, and their relative expression and putative functions were described.

**Results:**

A total of 5 female donors (2 healthy and 3 with RA) were included. A mean of 5,773 cells per donor were captured, resulting in a total of 23,094 high-quality cells included in this study. The samples comprised 5 major cell types: 2 distinct macrophage populations, a neutrophil population, and minor populations of both B and T cells. There were no statistically significant differences in proportion of cell types between samples from healthy donors and those from patients with RA or between baseline and postinfection timepoints. However, gene modules of significantly differentially expressed genes between groups revealed transcriptional differences between groups that were associated with neutrophil function, including NETosis and inflammatory responses.

**Conclusion:**

Immune cell proportions in donors with RA and in healthy donors are similar both before and after infection. However, transcriptional differences within lung neutrophils persist up to 30 days following respiratory infection.

## Introduction

Rheumatoid arthritis (RA) is a chronic degenerative autoimmune disease that is diagnosed primarily by the presence of anti–citrullinated protein antibodies and chronic inflammation and destruction of the joint synovium.^1^ The prevalence of RA in the United States is estimated to be between 0.6% to 1% of the population, with women being 2 to 3 times more likely than men to present with RA.^2^ RA pathologically modifies the tissue of additional organs beyond the synovium, including lung tissue. Clinically significant interstitial lung disease is present in 2% to 10% of patients with RA, and severity of RA symptoms predicts the likelihood of diagnosis of interstitial lung disease.^1^ Respiratory mucosal exposures to infection have been associated with the development and progression of RA.^1^ Patients with RA in turn have increased risk of respiratory infection.^1^

Neutrophils play a role in RA pathogenesis, generating citrullinated proteins and peptidyl arginine deiminases that further citrullinate proteins, and directly damage tissue.^3^ Neutrophils infiltrating the lungs also serve as a primary immune response to respiratory infection, contributing to a proinflammatory response and releasing neutrophil extracellular traps (NETs) that bind pathogens.^3^ Traditional single-cell RNA sequencing (scRNA-seq) excludes neutrophils, as they do not survive processing. However, by combining fixed scRNA-seq of sputum from the human lung with custom cell detection analysis, we recovered neutrophils in sputum from participants with RA and healthy participants before and 1 month following acute respiratory viral infection (ARVI). Our results suggest that enhanced inflammation is associated with neutrophils in patients with RA both at baseline and following infection.

## Results and discussion

**Fig 1:**
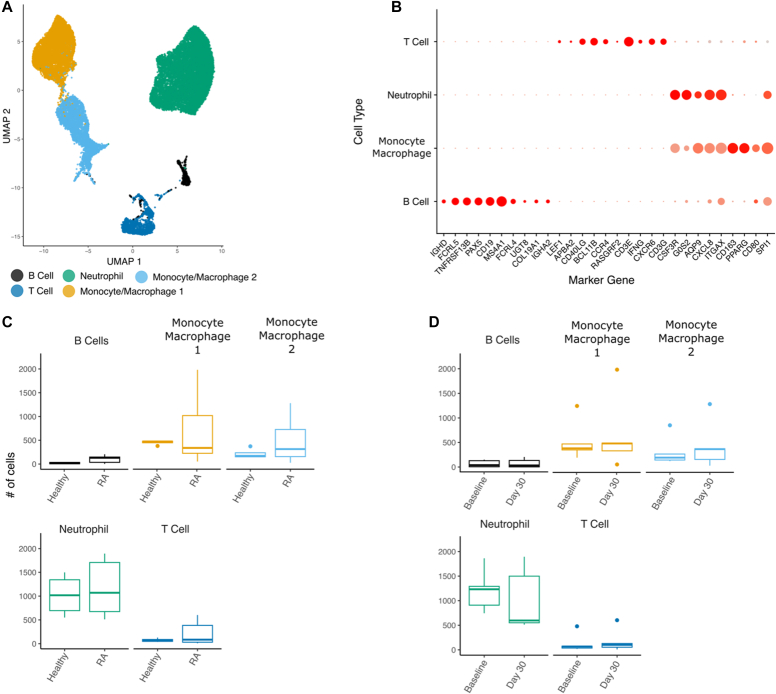
Immune cell changes in sputum in participants with RA and healthy participants before and after acute respiratory viral infection. **A,** Uniform Manifold Approximation and Projection (UMAP) reduction of gene expression of cells. Label annotation assigned automatically by Azimuth typing and manually via canonic markers. **B,** Dotplot matrix of marker gene expression for annotated clusters. Size of circles indicative of the proportion of cells within cluster positively expressing gene; color of dot represents the intensity of expression. **C** and **D,** Boxplots of cell proportions across study group (with RA vs healthy) and timepoint (all comparisons are nonsignificant; box lines denote median; whiskers 1.5 × interquartile range).

Cells collected from 5 female participants (2 healthy participants, 3 with RA) at 2 timepoints were included in this study, resulting in 23,094 high-quality cells retained following quality control (Table I). The participants with RA did not have high disease activity during the study period. Total cell recovery was variable, ranging from 2,167 to 10,485 cells per donor (median 3,313 cells). We performed Louvain clustering of cells and reduced the top 30 principal components of transcriptional variation into a Uniform Manifold Approximation and Projection (Fig 1, *A*). Reference mapping was successful in identifying all major cell types excluding neutrophils, which are not present in Azimuth lung references (see supplemental Methods in the Online Repository at www.jaci-global.org). We used canonic gene markers to both confirm reference-mapped cell labels and positively identify the neutrophil cluster (Fig 1, *B*). Levels of known neutrophil markers (colony stimulating factor 3 receptor [*CSF3R*], G0/G1 switch 2 [*G0S2*], aquaporin 9 [*AQP9*], and C-X-C motif chemokine ligand 8 [*CXCL8*]) were elevated in the distinct neutrophil cluster, allowing us to confidently identify this cell type. Macrophage and neutrophil populations dominated the immune cell composition of our samples, with a mean of 42.1% and 48.0% of all recovered cells, respectively (Table I). The macrophages formed 2 distinct subclusters: activated (macrophage 1) and homeostatic (macrophage 2) (see Table E1 in the Online Repository at www.jaci-global.org).

**Table I:** Participant demographics and cell counts

Participant, no.	Age, y	Sex	Race	Status	Receiving a DMARD	Receiving TNF-αi	Baseline to infection period (d)	Virus	Cells recovered, baseline (n)	Cells recovered, at 1 mo (n)	Cells recovered, total
1	36	F	White	Healthy	—	—	6	Rhinovirus	1,346	1,246	2,592
2	77	F	White	Healthy	—	—	118	SARS CoV-2	2,025	2,512	4,537
3	73	F	White	RA	Yes	No	99	Coronavirus 229E	1,566	601	2,167
4	51	F	White	RA	Yes	Yes	49	SARS CoV-2	4,589	5,896	10,485
5	52	F	Asian	RA	Yes	Yes	9	SARS CoV-2	1,717	1,596	3,313

*DMARD,* Disease-modifying antirheumatic drug; *F*, female; *SARS CoV-2*, severe acute respiratory syndrome coronavirus 2; *TNF-αi,* TNF-α inhibitor.

There were no statistically significant differences in the cell type proportions of immune cells between the samples from patients with RA and those from healthy participants (Fig 1, *C*). Additionally, the preinfection and postinfection samples did not demonstrate a shift in proportions of immune cell types, indicating relative stability of these populations over time. However, differentially expressed genes between both disease condition (from participants with RA vs from healthy participants) and timepoint (baseline vs day 30) were detected after pseudobulking transcript expression of the samples. Within the neutrophil compartment specifically, *ZNF107* and *ATP8B4* were differentially expressed at *P* < .05 between the samples from participants with RA and the samples from healthy participants across time, indicating that subtle differences within neutrophils were present at the single-cell level (see Fig E1 in the Online Repository at www.jaci-global.org).

**Fig 2:**
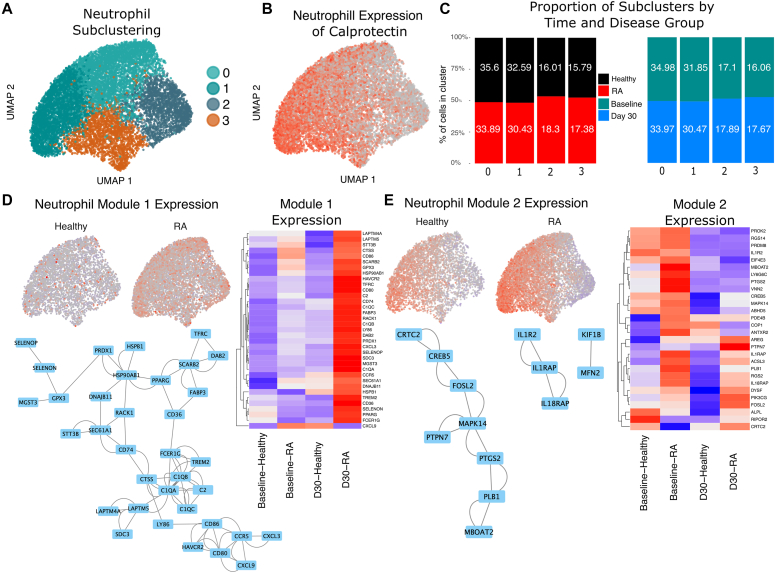
Differences in the neutrophil compartment reveal transcriptomic differences between participants with RA and healthy participants. **A,** Louvain clustering of neutrophils showing 4 subclusters. **B,** Calprotectin heterodimer (S100A8/S100A9) expression across neutrophils, as a proxy for degranulating activity. **C,** Proportioning of each study group (participants with RA and healthy participants) assigned to each neutrophil subcluster. Percentages within bars denote the contribution of that particular cluster to the group total (eg, healthy, with RA, baseline, and day 30), demonstrating an even distribution across clusters. **D,** Aggregate per-cell expression of genes contained within neutrophil module 1, showing greater expression of genes within RA cells. Heatmap of *z* scores of gene expression of most central genes within the module 1 network. Central genes were identified as having a stringDB confidence threshold greater than 700 and were selected by number of edges greater than 3. Network map of gene-gene interactions within module 1. **E,** Aggregate per-cell expression of genes contained within neutrophil module 2, Heatmap of *z* scores of gene expression of most central genes in module 2, and network map of gene-gene interactions within module 2.

Louvain clustering of neutrophils resulted in 4 clusters (Fig 2, *A*). Calprotectin expression (*S100A8/S100A9*) formed a gradient across these clusters, suggesting that granulizing activity plays a role in our neutrophil phenotypes (Fig 2, *B*). When reference-mapped against published data sets, the clusters correlated with known neutrophil populations, as labeled in prior literature^4^: clusters 0 and 1 correlated with extrafollicular trap–producing neutrophils expressing *CXCR1, CXCR2, ITGB2, NCF4, GCA, RGS2,* and *RNF149*; cluster 2 correlated with interferon-stimulated neutrophils with elevated expression of *MX1, OAS3, IFIT2, IFI44, ISG15, ISG20, IFITM2,* and *IFITM3*; and cluster 3 correlated with TNF-α– and nuclear factor-κB–stimulated inflammatory neutrophils with upregulated expression of the central neutrophil markers *G0S2, CXCL8, ITGAX, IL1B, NAMPT,* and *CCRL2* (see Figs E2-E4 in the Online Repository at www.jaci-global.org). These subclusters were evenly derived from the samples from both healthy participants and those with RA, as well as from the samples collected at both the preinfection and postinfection timepoints (Fig 2, *C* and see Figs E5 and E6 in the Online Repository at www.jaci-global.org), suggesting that the proportions of neutrophil subsets were neither distinct within participants with RA disease nor associated with a predisease or postdisease state.

At the single-cell level, the largest module (module 1) of coexpressed genes within neutrophils is broadly upregulated in patients with RA versus in healthy controls across the entire neutrophil population. This effect is driven by significantly higher expression values of module genes in the postinfection samples from participants with RA (*P* < 2.2e^–16^). The central genes within this module form an interacting network that includes the complement pathway as well as cytokine and proinflammatory genes (Fig 2, *D*).

Module 2 is more localized within neutrophil subsets, being highest in cluster 0 and lowest in cluster 2. The level of this module is elevated in participants with RA versus in healthy controls at baseline (*P* < 2.2e^–16^). The genes in this module reflect activation of the IL-1 cytokine pathway and neutrophil activation, maturation, and degranulation, including in markers such as *MAPK14* and *FOSL2*. Additionally, module 2 contains proinflammatory genes upregulated within the samples collected from donors with RA on day 30 versus in the samples collected from healthy donors on day 30, including several interleukin accessory proteins, implying that lingering signals of acute inflammation are still present specifically among donors with RA (Fig 2, *E*).

Neutrophils play an active role in both resolution of infectious disease and exacerbation and progression of RA, and they have attracted substantial attention for their role in progression of RA lung disease in murine scRNA models.^5^ There is a dearth of analogous research at single-cell resolution for neutrophils within the human lung in relation to RA and the respiratory environment. Here, we present the first scRNA analysis including neutrophils within the human lung for the purpose of investigating lingering transcriptional changes within this cell population following ARVI.

Both RA and ARVI have been shown to cause significant perturbations to the lung environment. Although the composition of immune cells infiltrating lung tissue has been shown to change during the acute phase of respiratory infection, here we have shown that the proportions of immune cell populations within the lung 30 days following ARVI are statistically indistinguishable from preinfection baselines in both participants with RA and healthy controls. Interestingly, despite this return to baseline cellular composition, subtle transcriptional differences can be detected between the baseline and postinfection samples in whole-sample pseudobulked analyses, suggesting that although the cell proportions have returned to baseline, transcriptional profiles are still modified 1 month after infection.

Single-cell analyses have identified modules of coexpressed genes that were significantly differentially expressed between participants with RA and healthy participants at both baseline and 30 days after infection. Module 1 primarily comprised proinflammatory genes that are active in patients with RA 30 days after infection but are relatively downregulated in all other groups. Many of these proinflammatory genes are those that would be expected to upregulate in response to infection; this includes *CXCL8*, *CXCL3,* and several complement pathway genes. Exclusively elevated in postinfection samples from participants with RA, this module suggests a failure to return to homeostasis in lungs from participants with RA following infection that is not mirrored in samples from healthy participants. Upregulation of *PPARγ* within this module further suggests transcriptional dysregulation. *PPARγ* is a negative regulator of inflammation, and upregulation of this gene is associated with resolution of inflammatory response. As levels of both *PPARγ* and its suppressive targets are upregulated in patients with RA 30 days after infection, it is likely that module 1 represents a proinflammatory module in response to infection that is dysregulated weeks after infection has been resolved.

The main limitation of our study is sample size. This study included samples from only 5 individuals, which limits the generalizability of this study. However, because the study gathered single-cell data from 23,094 high-quality cells, we believe that these data provide a reference for future studies and proof of concept for this technology. It is also important to note that this study included only female subjects, as a result of which the results may possibly be biased based on sex. The inclusion of only female subjects is nevertheless reflective of the strong female bias in RA prevalence.

Although healthy participants and participants with RA have similar immune compositions within sputum, patients with RA have upregulated neutrophil proinflammatory modules. These findings suggest that proinflammatory complement and chemokine signals may linger excessively in patients with RA following respiratory infection and may contribute to worsening outcomes in the lungs of patients with RA. Ongoing investigations, including the collection of additional scRNA sequencing data from fixed sputum samples, will further clarify the lingering transcriptional dysregulation of neutrophils within the RA lung.

## Disclosure statement

Supported by the 10.13039/100000002National Institutes of Health (grant U19AI167891).

Disclosure of potential conflict of interest: The authors declare that they have no relevant conflicts of interest.

## References

[1] Alunno A., Gerli R., Giacomelli R., Carubbi F. (2017). Clinical, epidemiological, and histopathological features of respiratory involvement in rheumatoid arthritis. BioMed Res Int.

[2] Gerosa M., De Angelis V., Riboldi P., Meroni P. (2008). Rheumatoid arthritis: a female challenge. Women’s Health (Lond.).

[3] Khandpur R., Carmona-Rivera C., Vivekanandan-Giri A., Gizinski A., Yalavarthi S., Knight J.S. (2013). NETs are a source of citrullinated autoantigens and stimulate inflammatory responses in rheumatoid arthritis. Sci Transl Med.

[4] Doni Jayavelu N., Liu A.H., Gaberino C., Freeman K., Lawrance M., Pribitzer S. (2025). Single-cell transcriptomic profiling of eosinophils and airway immune cells in childhood asthma. J Allergy Clin Immunol.

[5] Xue J., Nian M., Liang Y., Zhu Z., Hu Z., Jia Y. (2025). Neutrophil extracellular traps (NETs) are increased in rheumatoid arthritis-associated interstitial lung disease. Respir Res.

