# Supplementary material for: Neutrophils have altered response to acute respiratory viral infection in sputum of patients with rheumatoid arthritis
Source: J Allergy Clin Immunol Glob. 2026 Feb 26;5(3):100665. doi: 10.1016/j.jacig.2026.100665 (PMC12993894; doi:10.1016/j.jacig.2026.100665)
Supplement: Supplementary Figs and Tables [file mmc1.docx]

**Materials and Methods:***Study approval:* This study was approved by the Benaroya Research Institute Institutional Review Board (IRB#: IRB22-016). Written informed consent from each participant was received prior to participation.

*Enrollment/study design:* Adult participants greater than 18 years of age with rheumatoid arthritis (RA) were enrolled into this longitudinal study. Participants with RA were diagnosed based on 2010 ACR/EULAR criteria and could be treated with a disease-modifying anti-rheumatic drug (DMARD), including methotrexate, hydroxychloroquine, sulfasalazine or leflunomide, or TNF-α inhibitor. Participants on prednisone greater than 5mg per day or other immunosuppressants were excluded. Healthy control (HC) participants were screened to have no personal history or first-degree relatives with RA. Other exclusion criteria for both RA and HC participants included pregnancy, incarceration, current smoking or vaping, or chronic viral infection including HIV, Hepatitis C, or Hepatitis B.

*Sputum Induction:* Induced sputum was collected from participants using inhalation of nebulized 7% saline over the course of 15 minutes followed by an additional 15 minutes for sputum collection. This was performed while the patient was in an AeroMed ATC Sputum Induction Booth. Sputum samples were weighted and treated with treated with 0.1% DTT for 15 minutes at 37°C and filtered through a 40-micron cell strainer to obtain a single cell suspension. A maximum of one million cells were fixed using formaldehyde and 10X Genomics Fix and Perm Buffer (10X Genomics PN-2000517) per the manufacturers protocol. Cells were fixed for 24 hours and stored in the manufacturers recommended long-term storage buffer, including 10% glycerol, and stored at -80°C. Fixed samples were processed according using the Chromium Next GEM Single Cell Fixed RNA Sample Preparation Kit (10x Genomics). Briefly, fixed samples were thawed and hybridized overnight to one of four human probe sets (10x Genomics). Pooled samples were washed and loaded onto the Chromium X (10x Genomics) for partitioning into GEMs. Gene expression libraries were generated according to the Chromium Fixed RNA Profiling Reagent Kits for Multiplexed Samples user guide. Sequencing was carried out on a NextSeq 2000 sequencer, using a NextSeq 2000 P3 XLEAP-SBS flowcell (Illumina) with a target depth of 10,000 reads/cell.

*Analysis:* CellRanger v7 (10x Genomics) was used according to established protocols. CellRanger identifies empty GEMs by seeking an inflection point in unique molecular identifier (UMI) density counts in a rank plot of cell barcodes. In cases where transcriptionally sparse cells such as neutrophils are part of a complex sample containing other more transcriptionally dense cell types, this algorithm tends to exclude neutrophils entirely from called cells. To account for this, the raw, unfiltered barcodes were taken from CellRanger’s output, and filtered by use of emptyDrops, with a minimum threshold of 100 UMI forming the lower bounds of cell calls. GEMs below this threshold were considered to contain no cells and represented ambient RNA background. Cells exceeding 100 UMI were tested against this ambient distribution and excluded if they were not significantly distinct^[1]^.

Any cell containing more than 12.5% mitochondrial reads were excluded to account for dead/dying cells, and cells with greater than 6,000 features or 35,000 UMIs were excluded as likely multiplets of heterogenous and homogenous cell types respectively.

Cells were assigned labels using Azimuth reference mapping, with the lung v2 dataset providing a reference^2,3^. The lung v2 reference was constructed on un-fixed 10x scRNA and lacks any neutrophilic signal. Neutrophils were, therefore, identified utilizing canonical markers. The mean contribution of each cell type was calculated and tested via a Student’s t-test, with nonsignificant p-values > .05 after a Bonferroni correction. Cell types at label level 1 were subsequently analyzed individually in order to isolate cell-type specific signals between RA and HC. Neutrophils, specifically, were subsetted from the overall object and additionally clustered.

*Statistics:* Differentially expressed genes for HC/RA at both baseline and post-infection timepoints were calculated in each cell type by Wilcoxon Rank Sum tests as implemented in FindAllMarkers within the Seurat Package. A single-cell dataset containing these DEGs for all cells was constructed in monocle3 and gene modules of these DEGs were calculated via find_gene_modules according to established methods with a resolution parameter of .01. Each module was cross referenced via stringDB with a confidence of 700, representing high confidence of gene-gene interactions within this module. Z-scored expression of DEGs were visualized with ComplexHeatmap. A *p* value less than 0.05 was considered significant.

**References:**

**1** Lun, A. T. L., Riesenfeld, S., Andrews, T., Dao, T. P., Gomes, T., participants in the 1st Human Cell Atlas Jamboree, & Marioni, J. C. (2019). EmptyDrops: distinguishing cells from empty droplets in droplet-based single-cell RNA sequencing data. Genome biology, 20(1), 63. https://doi.org/10.1186/s13059-019-1662-y

**2** Hao, Y., Hao, S., Andersen-Nissen, E., Mauck, W. M., 3rd, Zheng, S., Butler, A., Lee, M. J., Wilk, A. J., Darby, C., Zager, M., Hoffman, P., Stoeckius, M., Papalexi, E., Mimitou, E. P., Jain, J., Srivastava, A., Stuart, T., Fleming, L. M., Yeung, B., Rogers, A. J., … Satija, R. (2021). Integrated analysis of multimodal single-cell data. Cell, 184(13), 3573–3587.e29. <https://doi.org/10.1016/j.cell.2021.04.048>

**3** Sikkema, L., Ramírez-Suástegui, C., Strobl, D. C., Gillett, T. E., Zappia, L., Madissoon, E., Markov, N. S., Zaragosi, L. E., Ji, Y., Ansari, M., Arguel, M. J., Apperloo, L., Banchero, M., Bécavin, C., Berg, M., Chichelnitskiy, E., Chung, M. I., Collin, A., Gay, A. C. A., Gote-Schniering, J., … Theis, F. J. (2023). An integrated cell atlas of the lung in health and disease. Nature medicine, 29(6), 1563–1577. https://doi.org/10.1038/s41591-023-02327-2

**Supplemental Figure 1:** Differentially expressed genes via pseudobulking of neutrophils in these samples. Log-fold changes are relative to the RA condition.

**Supplemental Figure 2:** Selected genes of referenced maps cluster distributed across query clusters from this dataset.

**Supplemental Figure 3:** UMAP of our cell dataset reference mapped to Jayavelu et al, 2025, demonstrating three main reference cluster (Neut 0, Neut1, and Neut3) common between our dataset. Gray points are cells that did not assign to any reference label at greater than .75 likelihood.

**Supplemental Figure 4:** Proportion assignments of Jayavelu et al 2025 reference clusters across our query clusters at highly confident cells (>.75 likelihood), showing an uneven distribution of reference labels.

**Supplemental Figure 5:** Cell proportions broken out by timepoint and disease state showing no significant variation of cell type across sample categories.

**Supplemental Figure 6:** Boxplot of proportion of each sample category across all four neutrophil clusters.

**Supplemental table 1: Differentially Expressed Activation Genes in Macrophage 1**

| **gene** | **p_val** | **avg_log2FC** | **pct.1** | **pct.2** | **p_val_adj** |
| --- | --- | --- | --- | --- | --- |
| IL1A | 0 | 1.22741057 | 0.693 | 0.271 | 0 |
| IL17RB | 0 | 3.13469584 | 0.422 | 0.038 | 0 |
| IL7 | 4.78E-117 | 1.36738389 | 0.312 | 0.105 | 1.89E-112 |
| IL11RA | 2.43E-109 | 1.10989176 | 0.33 | 0.125 | 9.61E-105 |
| IL6 | 6.27E-106 | 0.94456261 | 0.301 | 0.109 | 2.48E-101 |
| IL16 | 1.17E-99 | 0.29976445 | 0.699 | 0.434 | 4.65E-95 |
| IL17RC | 2.81E-83 | 0.59938191 | 0.583 | 0.346 | 1.11E-78 |
| IL12RB1 | 4.92E-51 | 0.16730336 | 0.654 | 0.444 | 1.95E-46 |
| IL37 | 3.06E-18 | 1.44099045 | 0.025 | 0.002 | 1.21E-13 |
| IL17D | 0.00102877 | 0.29371655 | 0.013 | 0.006 | 1 |
| TGFBR2 | 8.78E-230 | 0.72593674 | 0.923 | 0.699 | 3.48E-225 |
| TGFBRAP1 | 7.49E-27 | 0.14105269 | 0.597 | 0.427 | 2.97E-22 |
| CD163 | 1.10E-93 | 0.28947654 | 0.982 | 0.781 | 4.37E-89 |
